# Design and implementation of the canadian kidney disease cohort study (CKDCS): A prospective observational study of incident hemodialysis patients

**DOI:** 10.1186/1471-2369-12-10

**Published:** 2011-02-16

**Authors:** Aminu K Bello, Ravi Thadhani, Brenda Hemmelgarn, Scott Klarenbach, John Gill, Christopher Chan, Deborah Zimmerman, Daniel Holmes, George Cembrowski, Dawn Opgenorth, Rafael Sibrian, Mohammad Karkhaneh, Sophanny Tiv, Natasha Wiebe, Marcello Tonelli

**Affiliations:** 1 Department of Medicine, University of Alberta, Edmonton, Alberta, Canada; 2 Department of Medicine, Massachusetts General Hospital, Boston, USA; 3 Department of Medicine, University of Calgary, Calgary, Alberta, Canada; 4 Division of Nephrology, University of British Columbia, Vancouver, BC, Canada; 5 Division of Nephrology, University Health Network, University of Toronto, Toronto, Ontario, Canada; 6 Department of Medicine, University of Ottawa, Ottawa, Ontario, Canada; 7 Department of Pathology & Laboratory Medicine, University of British Columbia, Vancouver, BC, Canada; 8 Department of Laboratory Medicine & Pathology, University of Alberta, Edmonton, Alberta, Canada

## Abstract

**Background:**

Many nephrology observational studies use renal registries, which have well known limitations. The Canadian Kidney Disease Cohort Study (CKDCS) is a large prospective observational study of patients commencing hemodialysis in five Canadian centers. This study focuses on delineating potentially reversible determinants of adverse outcomes that occur in patients receiving dialysis for end-stage renal disease (ESRD).

**Methods/Design:**

The CKDCS collects information on risk factors and outcomes, and stores specimens (blood, dialysate, hair and fingernails) at baseline and in long-term follow-up. Such specimens will permit measurements of biochemical markers, proteomic and genetic parameters (proteins and DNA) not measured in routine care. To avoid selection bias, all consenting incident hemodialysis patients at participating centers are enrolled, the large sample size (target of 1500 patients), large number of exposures, and high event rates will permit the exploration of multiple potential research questions.

**Preliminary Results:**

Data on the baseline characteristics from the first 1074 subjects showed that the average age of patients was 62 (range; 50-73) years. The leading cause of ESRD was diabetic nephropathy (41.9%), and the majority of the patients were white (80.0%). Only 18.7% of the subjects received dialysis in a satellite unit, and over 80% lived within a 50 km radius of the nearest nephrologist's practice.

**Discussion:**

The prospective design, detailed clinical information, and stored biological specimens provide a wealth of information with potential to greatly enhance our understanding of risk factors for adverse outcomes in dialysis patients. The scientific value of the stored patient tissue will grow as new genetic and biochemical markers are discovered in the future.

## Background

More than 1 million patients with end-stage renal disease (ESRD) receive renal replacement therapy (RRT) worldwide in the form of dialysis or transplantation, and this number is projected to reach 2.2 million by the year 2030 [1-3]. In Canada, about 21,000 individuals require hemodialysis (HD) treatment for ESRD, with an expected annual growth of 7% [4]. Developed nations spend about 2-6% of their total healthcare budget for the provision of RRT to their ESRD population, which usually represents only about 0.02-0.6% of their total population [5,6]. In addition, mortality for ESRD patients is 10 to 1000 times greater than in age matched controls with normal kidney function, and ESRD is also associated with very low quality of life [7-10]. The high costs of care for RRT, adverse outcomes and the increasing burden of ESRD make it a major global public health issue.

The widespread use of RRT to prolong life for people with ESRD has been one of the major advances in medicine [8]. Nonetheless, the limitations of dialysis for treatment of ESRD remain problematic. As the number of patients treated grows, the costs, and morbidity and mortality remains high despite technical and scientific improvements [8,11,12]. Despite intensive investigation, our knowledge of factors that mediate uremic complications among people treated with dialysis remains inadequate[8,13].

Observational studies are important sources of information to close knowledge gaps in every clinical discipline. However, most observational studies in ESRD have collected either good quality information on a small number of individuals, or poorer quality data on a much larger number of individuals. Many existing observational data are derived from renal registries such as the United States Renal Data Systems (USRDS) [5], European Renal Association - European Dialysis and Transplant Association (ERA-EDTA) [14], and Australia and New Zealand Dialysis and Transplant (ANZDATA) [15]; or from prospective studies such as the Dialysis Outcomes and Practice Pattern Study (DOPPS) [16], the choices for Healthy Outcomes in Caring for ESRD (CHOICE) Study [17], and European Uraemic Toxins (EUTox) Work Group database [13]. Although these studies have made substantial contributions to our knowledge about uremia and its optimal treatment, they may have limitations including suboptimal statistical power, lack of detail in data collection, or inability to store a comprehensive battery of biological specimens for future analyses.

The Canadian Kidney Disease Cohort Study (CKDCS) is a large prospective observational study of patients commencing dialysis in five Canadian centers serving ethnically mixed populations (Vancouver, Calgary, Edmonton, Ottawa, and Toronto). This novel study focuses on identifying the potentially reversible determinants of the major complications associated with dialysis in an incident cohort of adult patients.

The key research objectives for the CKDCS are:

1. To assemble a prospective cohort of incident Canadian hemodialysis patients and associated database which contains research-quality information on patient characteristics, comorbid diseases and routine and novel laboratory markers and genetic data.

2. To utilize data from the cohort to test specific hypotheses about the independent relation between biochemical and genetic markers and cardiovascular disease (CVD) risk and outcomes such as mortality, hospitalization and quality of life measures

3. To rapidly capture information on the characteristics and health history of incident hemodialysis patients, using a structured patient interview and review of medical records.

4. To operate a specimen management and storage system so that diverse specimens (blood, dialysate, fingernails, hair) can be collected at dialysis initiation and eventually be assayed by new biochemical and genetic assays.

5. To make the data available to other academic investigators and collaborators if required.

## Methods

In order to have the most representative registry, the CKDCS enrolls all incident adult (≥18 years of age) hemodialysis patients from the five participating renal programs. The sole exclusion criterion is failure to provide informed consent.

### Recruitment

All subjects commencing chronic hemodialysis (HD) are approached by study staff within 8 weeks of commencing dialysis therapy. A variety of options for participating in the study are offered, depending on the interests of the patient (Appendix 1).

### Medical Data Collection

Consenting participants undergo a structured interview to collect detailed data on demographic characteristics, medical history, social history and satisfaction with care. Information from the clinical record is used to supplement the history. At the time of the first annual follow-up visit, study subjects in Edmonton, Calgary and Vancouver independently complete a Kidney Disease Quality of Life Questionnaire (KDQOL), Health Utility Index questionnaire (HUI version 3) and an Attitudes Toward Transplantation Questionnaire. To avoid unnecessary expenditures on laboratory tests, routine test results (complete blood count [CBC], urea, creatinine, electrolytes, calcium, phosphate, parathyroid hormone [PTH]) are captured in the study database using either a direct data link or entered manually by study personnel (Table 1).

**Table 1:** Data Collection Synopsis

Category	Data Collected	Baseline	Followup
Background and demographic information	Age, sex, race, dialysis start date, education, employment status, postal code (to allow linkage to census data), provincial health card number (for linkage), hospital unit number, social support	X	
Medical history	Cause(s) of ESRD, tobacco use/dose, coronary disease, chronic heart failure, previous arrhythmia, cerebrovascular disease, peripheral vascular disease, diabetes mellitus, pulmonary disease, neurologic disease, psychiatric disease, musculoskeletal disease, gastrointestinal and hepatic diseases, cancer, eye disease, hepatitis, HIV, family history of premature CAD in first degree relatives	X	
Pre-ESRD treatment	Nephrologist visit, interval between first nephrology visit and ESRD, vascular access, relevant labs (Hb, Ca, PO_4_, HCO_3_, albumin)	X	
Vital signs	BP, HR, weight, height	X	X
Dialysis prescription	Time, dialyzer, dialysate composition, blood flow, Bioimpedance measurements	X	X
Functional Status	Specific study questionnaire, Health Utilities Index	X	X
Laboratory data	Routine laboratory data from dialysis monthly bloodwork	X	X
	Frozen blood for biochemistry		
	Frozen plasma for special tests and proteomic studies (will not be thawed or refrozen)		
	Frozen blood for DNA/RNA		
	Frozen dialysate		
Excess/deficiency of trace elements	Source water collection from home and dialysis unit	X	X
Residual renal function	Timed urine collection for urea/creatinine	X	X
Medications	List of medications and dose	X	X
Vascular access history	Type and location of current access, number of prior temporary and permanent accesses	X	X
Vascular access events	Type, location at start and end of interval; all changes in access status; procedures, creation/placement	X	X
Hospitalizations	Dates, diagnosis, and procedures for each interval hospitalization		X
Interval status	Dialysis status at end of reporting interval, date and cause of death		X
Determinants of Transplant referral	Patient and physician attitudes toward transplantation, intervals from transplant referral to transplant activation.	X	X
Quality of life	Kidney Disease Quality of Life Instrument (KDQOL)	X	X
Satisfaction with care	Specific study questionnaire		X
Coronary artery calcification/Cardiac geometry	Coronary CT scan/Cardiac MRI	X	

BP = Blood pressure, CAD = Coronary Artery Diseas, ESRD = Endstage renal disease, HR = Heart rate, KDOOL = Kidney Disease Quality of Life Instrument, Hb = Haemoglobin, Ca = Calcium, PO4 = Phosphate, HCO_3_, = Bicarbonate

Study visits are conducted at baseline, month 6, year 1, year 2, year 3, year 4, year 5 and then every 5 years thereafter. The baseline study visit is conducted within 8 weeks of initiating chronic dialysis. Follow-up visits are completed ± 2 month of the scheduled visit date (Table 2).

**Table 2:** Study Procedure Schedule

Procedures	Study Visits Baseline	Month 6	Month 12	Month 18	Year 2 and yearly
Consent	X				
Subject interview	X	X	X		X
Medical record review	X	X	X		X
Coronary CT (separate consent form)	X				
Sample collection (blood, dialysate/effluent, finger/toe nails, hair)	X	X	X		X
Home tap water collection	X	X			
Genetic sample collection (separate consent form)	X				
Control genetic sample collection (separate consent)	X				
Bioimpedance measurement	X	X	X	X	X
KDQOL	X	X	X		X
Attitudes toward Transplant			X		

CT = Computed tomography, KDOOL = Kidney Disease Quality of Life Instrument

Whenever necessary, a translator is present during the consent and interview procedures. Quality of Life questionnaires (which must be completed by the study subject without assistance) are available in English, French, Chinese and Spanish and may be completed by hand or electronically.

### Sample Collection and Processing

Blood, dialysate effluent, fingernail and hair samples are obtained at baseline, 6 months, 12 months and at each study visit thereafter, according to standard protocols. Blood samples are immediately divided into multiple aliquots, from which plasma, DNA, RNA and/or cells are extracted and separately stored. Dialysate specimens are aliquoted to allow future measurement of genetic and cellular material. Specimens are frozen at -80 degree Celcius. Specimens are sent to the Canadian Biosample Repository in Edmonton and are labeled using a unique study identifier to maintain confidentiality.

### Source Water Collection

Source water used for dialysate is collected in triplicate four times annually from each dialysate unit (including satellite dialysis locations). Patients are also asked to bring a sample of their drinking water from home on two separate days during one week at baseline and month 6. Source water specimens are shipped to the study laboratory and frozen for future analysis.

### Coronary CT and Cardiac MRI

A subset of 500 subjects will undergo imaging to determine the presence and extent of Coronary Artery Calcification (CAC) and Cardiac geometry including Left Ventricular Hypertrophy (LVH). CAC will be assessed by multi-slice computed tomography at two points during the study: baseline (within 3 months of dialysis initiation) and 12 months after dialysis initiation. All CT scans will be reported locally by an experienced reader for the presence, distribution and amount of CAC, using dedicated software (manufacturer dependent). Calcium mass, plaque volume and Agatston score will be calculated [18].

To ensure uniformity and high quality image acquisition, all sites must: (1) use ≥64-slice multidetector Computed Tomography Scan (MDCT) technology or better (2) led by level 3 accredited cardiologist/radiologist. Before the start of the study, each site will send ten anonymized CAC reports and data sets to the core lab for assessment of image quality/data transfer/reproducibility of results. For sites demonstrating excessive variation, the core lab will review the site protocol/analysis tools and work with the site to reach the standard needed.

Cardiac geometry (including LVH) will be assessed by cardiac magnetic resonance imaging (CMRI). Scanning is performed at baseline and at 12 months after dialysis initiation in these 500 participants. Left ventricular (LV) mass, and change in LV mass are analyzed as both quantitative traits and dichotomous end-points. All CMRI scans will be analyzed using dedicated software (manufacturer dependent) and reported locally by an experienced reader. Absolute and normalized to body surface area (BSA) left ventricular muscle mass, end diastolic blood pool volume, end systolic blood pool volume, ejection fraction, cardiac output and stroke volume will be calculated. Results forwarded to the core lab together with image data. To ensure uniformity and high quality image acquisition; all sites must: (1) use 1.5T CMR sequences (2) led by a level 3 CMRI certified radiologist/cardiologist. Before the start of the study, each site will send ten anonymized LV function data sets and results to the core lab for assessment of image quality/transfer capability and reproducibility of results. For sites demonstrating excessive variation, the core lab will review the site protocol/analysis tools and work with the site to reach the standard needed. An additional two studies will be requested to ensure the site fulfils all requirements.

### Bacteremia Screening

All participating renal programs perform ongoing surveillance of bacteremia in HD patients as an infection control initiative. Renal programs routinely record the date and microbiological species associated with positive blood cultures, as well as patient-specific information such as health card number and demographic data. Data on the management of bacteremia are also collected including: whether antibiotics were prescribed, antibiotic type/duration, whether catheter removal/exchange occurred and whether the event was resolved. Recurrent bacteremia is defined as a repeat positive blood culture with the same species within 6 months of the index event, according to current guidelines [19]. Periods of follow-up and any hospitalizations for infection or infectious complications within 2-8 weeks of the index culture are recorded.

### Death Adjudication

Study subjects who die are identified by monthly follow-up by study coordinators; date of death is obtained from the renal programs. A prospectively defined method is used to ascertain the cause of death as previously published [20,21]. A standardized form is completed by a study coordinator after obtaining the date of death. The forms are supplemented by information provided by the patient's physicians and next of kin where appropriate. This form, together with copies of the medical record, discharge summaries, autopsy/coroner's report (if available) are obtained and sent to two investigators for independent classification of the cause of death. On occasions when the cause of death differs between adjudicators, the site investigator acts as the tie breaker.

### Additional Data Sources

**Figure 1:**
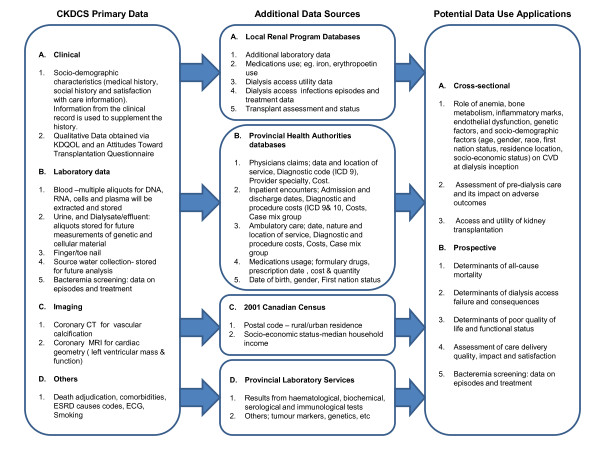
**Data sources utilized by linking patient identifiers for elaborate analysis of outcomes and their determinants from the primary data collected in the study, and that obtained from additional sources (Abbreviations: CKDS = Canadian Kidney Disease Cohort Study, CT = Computed tomography, CVD = Cardiovascular disease, ECG = Electrocardiogram, ESRD = Endstage Renal Disease, MRI = Magnetic Resonance Imaging, KDQOL, Kidney Disease Quality of Life)**.

Whenever possible, other data sources are utilized by linking patient identifiers for analysis of outcomes and their determinants (Figure 1). All data linkages are done to ensure the confidentiality of study subjects. Security measures will include: the encryption of subject identifiers and corresponding data, transfer of encrypted data on password protected storage media shipped by courier or through secure FTP sites. The following data sources may be utilized:

#### Local Renal Program Databases

Contain detailed information on pre-dialysis, dialysis and kidney transplant patients. Data linkage will provide information on medication use, laboratory results, bacteremia incidence and management, and access blood flows.

#### Provincial Health Authorities Databases

The use of administrative data sources permit determination of disease incidence and prevalence using validated algorithms for common medical conditions such as hypertension [22], diabetes [23,24], acute myocardial infarction [24], congestive heart failure [25] and stroke [26], as well as assessment of clinical, health outcomes and costing information [27]. In addition to routine demographic information and dates of death, some administrative databases include information to permit the assessment of Aboriginal ethnicity, socio-economic status, and a six-digit postal code which enables unique geographic information system (GIS) analyses to be performed [27]. Hospitalization information via diagnostic and procedure codes using ICD-9-CM and ICD-10) is available [27].

#### Canadian Census

Census data includes the population counts of each geographic area, as well as information, for example, on whether it is in a rural location (Statistics Canada definition)[28], average income, education levels, etc. The six-digit residential postal code for each subject is linked to these data using the Postal Code Conversion file.

#### Provincial Laboratory Services

Computerized provincial databases are used to obtain laboratory data recorded outside of the renal programs.

### Study Data Management

Study data are stored in a Microsoft SQL database created specifically for this project. Wherever possible, free text fields, blank values and open-ended questions have been eliminated from the database. "Not available" and "not applicable" values are permitted. Data from enrolled subjects are either entered directly into the database using a Microsoft Access interface through dedicated, password controlled laptop computers or are first collected on paper forms. All paper forms identify subjects exclusively by initials and study numbers. Quality assurance reports are generated on a monthly basis for the purposes of ensuring standard and compliance with the study protocol. Working copies of the database used to perform analyses are stripped of uniquely identifying information such as provincial health care number.

### Ethics Approval

The institutional review boards at the participating centers approved the study, and it is conducted according to the Helsinki declaration for medical research in humans.

### Planned Analyses

The sample size and large number of exposures together with the high event rate will permit the exploration of multiple potential research questions. In addition, the availability of stored blood samples will allow the testing of hypotheses raised by data which becomes available in the future. The target sample size is 1500 subjects.

### Potential Use of Study Data

This study will provide a combination of cross-sectional and prospective clinical data and biological specimens of huge potential for conduct of high quality observational studies and clinical trials (Figure 1).

Potential Applications of the Cross-sectional data:

1. Investigating the role of anemia, bone metabolism, inflammatory markers, endothelial dysfunction, genetic factors, and socio-demographic factors (age, gender, race, first nation status, residence location, socio-economic status) on CVD at dialysis inception

2. Assessment of pre-dialysis care and its impact on adverse outcomes

3. Access and utility of kidney transplantation

Potential Applications of the Prospective data:

1. Determinants of all-cause mortality

2. Determinants of dialysis access failure and consequences

3. Determinants of poor quality of life and functional status

4. Assessment of care delivery quality, impact and satisfaction

5. Bacteremia screening: data on episodes and treatment

6. Potential for biomarker discovery and validation using array technology

### Preliminary Results

Data on the baseline characteristics of the first 1074 subjects enrolled are presented in Table 3. The average age of patients was 62 (range; 50-73) years. The leading cause of ESRD was diabetic nephropathy. The majority of patients enrolled to date were white. Overall 86.5%, 50.7%, 46.7%, 20.2% and 12.7% patients had a history of hypertension, diabetes mellitus, coronary artery disease, congestive heart failure and cerebrovascular disease respectively. Only 15.7% of the study subjects were current smokers. The predominant dialysis access in use was tunneled central venous catheter (60.4% of the cohort). The baseline laboratory parameters (median [interquartile range]) were albumin: 34 (30-37) g/L, calcium: 2.2 (2.0-2.3) mmol/L, phosphate: 1.6 (1.2-1.9)mmol/L, PTH: 26.2 (10.7-45.8.3) pmol/L, hemoglobin: 99 (89-109) g/L, ferritin: 252 (136-472) ug/L, and transferrin saturation: 24 (17-31)%. The average Kt/V was 1.4 (1.2-1.7) %. Only 18.7% of the subjects received dialysis in a satellite unit, and over 80% lived within 50 km of the nearest nephrologist.

**Table 3:** Preliminary data on baseline patients' characteristics

	Cohort patients, N(%)
**N**	1074
Age*	62 (50, 73)
**Male**	662 (62)
**Race**	1074
White	813 (76)
Aboriginal	81 (8)
East Asian	47 (4)
South Asian	42 (4)
Other	91 (8)
**Marital status**	937
Married	581 (62)
Single	163 (17)
Divorced	104 (11)
Widow	89 (9)
BMI, kg/m^2^*	26 (22, 31)
**Cause of ESRD**	916
Diabetic nephropathy	380 (41.5)
Glomerulonephritis	154 (16.8)
Hypertensive/ischemic renal disease	68 (7.4)
Polycystic kidney disease	49 (5.3)
Other	265 (28.9)
Comorbidity	
Arrhythmia	214 (20.9)
Cancer	156 (14.4)
Cerebrovascular disease	130 (12.7)
Congestive heart failure	202 (20.2)
Chronic lung disease	125 (12.7)
Claudication	131 (13.3)
Coronary disease	481 (46.7)
Dementia	28 (2.8)
Diabetes mellitus	515 (50.7)
Diabetic retinopathy	224 (23)
HBV	30 (2.9)
HCV	39 (3.8)
HIV	2 (.2)
Hypertension	785 (86.5)
Pericarditis	27 (2.7)
Peripheral neuropathy	239 (24.2)
Peripheral vascular disease	209 (20.6)
Prior cardiac arrest	20 (2)
Psychiatric illness	148 (15)
Seizure disorder	34 (3.4)
**ECG**	310 (28.7)
Normal	61 (5.6)
Abnormal	142 (13.1)
No result	107 (9.9)
**ECHO**	320 (29.6)
Normal	14 (1.3)
Abnormal	50 (4.6)
No result	256 (23.7)
Current Smoker	154 (15.7)
	
**CLINICAL MANAGEMENT**	
Satellite HD unit^€^	201 (18.7)
Type of access	510
AVF	138 (27.1)
PTFE	10 (2)
Non tunnel CVC	42 (8.2)
Tunnel CVC	308 (60.4)
	
LABORATORY MEASURES*	
Albumin, g/L	34 (30, 37)
Calcium, mmol/L	2.2 (2, 2.3)
Calcium X phosphate, mmol^2^/L^2^	3.6 (2.9, 4.5)
Creatinine, μmol/L	534 (424, 682)
Ferritin, μg/L	252 (136, 472)
Hemoglobin, g/L	99 (89, 109)
Phosphate, mmol/L	1.6 (1.2, 1.9)
PTH, pmol/L	26.2 (10.7, 45.8)
Transaturation, %	24 (17, 31)
Urea, mmol/L	18.6 (14.6, 23.5)
Kt/V	1.4 (1.2, 1.7)
	
Distance to Nephrologist, km	482
≤50	387 (80.3)
51 - 100	43 (8.9)
101 - 200	30 (6.2)
> 200	33 (6.8)

*Median (interquartile range)

^€^all clinics outside of Edmonton, Calgary and Vancouver considered satellite unit

HD Hemodialysis; BMI body mass index; ESRD end-stage renal disease; HBV Hepatitis B virus; HCV Hepatitis C virus; HIV Human immunodeficiency virus; ECG electrocardiogram; ECHO echocardiogram; AVF Arterial Venous Fistula; PD Peritoneal dialysis; PTFE Polytetrafluoroethylene; CVC Central venous catheters; PTH parathyroid hormone

## Discussion

The CKDCS uses a unique study design to assemble a cohort of subjects recruited at hemodialysis inception in Canada to prospectively collect detail clinical and laboratory data as well as novel biochemical and genetic markers. While clinical trials are considered best evidence to guide clinical care and policy, this study will inform important knowledge gaps for several reasons [29]. First, observational studies are important sources of hypothesis-generating data about chronic diseases and their treatments [30]. Second, collecting good quality observational data may reduce the risk of incorrect inferences [21,31], based on retrospective studies or less detailed registry data [4,5,8,13,16]. Third, comprehensive high quality observational studies of dialysis patients remain an important research focus [21,31].

An ideal cohort study in ESRD should consist of a well-characterized population of incident patients to avoid prevalence-incidence bias [29,30,32]. The CKDCS involves patients recruited at inception of dialysis with meticulous data collation, and monitoring for outcomes. The study will be large enough to allow adequate power to explore covariates simultaneously, and have sufficient variability in patient and treatment characteristics to allow exploration of the associations between these characteristics and various outcomes under investigation. The study will collect and quantify known covariates that might potentially affect relevant outcomes in dialysis patients (death, hospitalizations, access failure, quality of life, cost), and employ an accurate, unbiased method of ascertaining such outcomes.

The additional strength and novelty of this study is the collection and storage of materials (blood, dialysate, fingernails, hair, etc) so that new risk factors such as the impact of trace elements on outcomes can be measured and studied in the future as they arise. The storage of genomic DNA and plasma for future analyses is a strength given the emerging importance of genetic and proteomic analysis [33-35]. This has great potential for biomarker discovery and validation using array technology in future.

These unique characteristics make this study novel, topical and an extension of the existing observational databases on ESRD population. The largest such database is the USRDS, which although extremely useful, derives much of its information from billings data, which has significant limitations [5]. For example, medical professionals do not generally enter data themselves, and the information collected is chiefly limited to the elements required for Medicare reimbursement. In general, quantified risk factors and information on laboratory results and medication use are not available. More recently, the DOPPS has sought to remedy some of these aforementioned limitations, but has been limited by lack of a biobanking platform and prospective imaging studies [16].

In conclusion, the CKDCS will capitalize on the unique demographic and multiethnic characteristics of the Canadian dialysis population, a supportive Canadian regulatory climate, and the established and productive collaborations between Canadian nephrology investigators to address the limitations of existing databases. It will also provide novel information on determinants of adverse outcomes in hemodialysis patients, because of its unique combination of detailed prospectively collected information and the availability of stored biological specimens. This will provide new platforms for clinical and translational observational studies and clinical trials among dialysis patients in the future.

## List of Abbreviations used

ANZDATA: Australia and New Zealand Dialysis and Transplant; BSA: Body Surface Area; CAC: Coronary Artery Calcification; CHOICE: Choices for Healthy Outcomes in Caring for ESRD; CKDCS: Canadian Kidney Disease Cohort Study; CMRI: Cardiac Magnetic Resonance Imaging; CBC: Complete blood count; CT: Computed tomography; CVD: cardiovascular disease; DOPPS: Dialysis Outcomes and Practice Pattern Study; ERA-EDTA: European Dialysis and Transplant Association; ESRD: End-stage renal disease; EUTox: European Uraemic Toxins; GIS: Geographic Information System; HD: hemodialysis; HUI: Health Utility Index questionnaire; KDQOL: Kidney Disease Quality of Life Questionnaire; LVH: Left Ventricular Hypertrophy; MDCT: multidetector Computed Tomography Scan; PTH: Parathyroid hormone; RRT: Renal Replacement Therapy; USRDS: United States Renal Data Systems.

## Competing interests

The authors declare that they have no competing interests.

## Authors' contributions

All authors have made substantial contributions to the development of the manuscript, and have all been involved in revising it for important intellectual content and approved the final version.

## Appendix 1: Study Consent Pathway

All subjects wishing to participate in the study are required to sign the Main CKDCS consent form which permits collection of health history and/or clinical laboratory data at baseline and during follow-up and will allow future analysis of stored blood specimens for non-genetic laboratory assays to examine "factors that influence the cause or treatment of heart or kidney disease".

1. **Genetic Sample Consent **- The Genetic Sample Consent form will permit proteomic and genetic testing (plasma and DNA) from stored peripheral blood and other available biological samples such as tissue, to examine the same research questions.

Both consent forms will explicitly state that:

• data and specimens may be obtained and used after the death of the subject

• provincial and national databases can be accessed to provide obtain other information about their health

2. **Control Genetic Sample consent **- Whenever possible, control blood samples are collected from a family member or acquaintances of the study subject. These samples are used as controls for the genetic analyses. Eligible family members or acquaintances will include those who do not have kidney disease and are willing to provide samples with the understanding that they are used in an anonymous fashion as the control in genetic analyses. The same safeguards are taken to protect the privacy of these control subjects, as is taken for participants treated with dialysis.

3. **CT consent **- The Coronary CT consent permits a multi-slice coronary CT scan at baseline.

## Pre-publication history

The pre-publication history for this paper can be accessed here:

http://www.biomedcentral.com/1471-2369/12/10/prepub
